# Oxfordshire community stroke project classification improves prediction of post-thrombolysis symptomatic intracerebral hemorrhage

**DOI:** 10.1186/1471-2377-14-39

**Published:** 2014-03-01

**Authors:** Sheng-Feng Sung, Solomon Chih-Cheng Chen, Huey-Juan Lin, Chih-Hung Chen, Mei-Chiun Tseng, Chi-Shun Wu, Yung-Chu Hsu, Ling-Chien Hung, Yu-Wei Chen

**Affiliations:** 1 Division of Neurology, Department of Internal Medicine, Ditmanson Medical Foundation Chia-Yi Christian Hospital, Chiayi City, Taiwan; 2 Min-Hwei College of Health Care Management, Tainan, Taiwan; 3 Department of Medical Research, Ditmanson Medical Foundation Chia-Yi Christian Hospital, Chiayi City, Taiwan; 4 Department of Neurology, Chi-Mei Medical Center, Tainan, Taiwan; 5 Department of Cosmetic Science, Chia Nan University of Pharmacy and Science, Tainan, Taiwan; 6 Department of Neurology, College of Medicine, National Cheng Kung University, Tainan, Taiwan; 7 Landseed Hospital, Tao-Yuan County, Taiwan; 8 Department of Neurology, Landseed Hospital, Tao-Yuan County, Taiwan; 9 Department of Neurology, National Taiwan University Hospital, Taipei, Taiwan

## Abstract

**Background:**

The Oxfordshire Community Stroke Project (OCSP) classification is a simple stroke classification system with value in predicting clinical outcomes. We investigated whether and how the addition of OCSP classification to the Safe Implementation of Thrombolysis in Stroke (SITS) symptomatic intracerebral hemorrhage (SICH) risk score improved the predictive performance.

**Methods:**

We constructed an extended risk score by adding an OCSP component, which assigns 3 points for total anterior circulation infarcts, 0 point for partial anterior circulation infarcts or lacunar infarcts. Patients with posterior circulation infarcts were assigned an extended risk score of zero. We analyzed prospectively collected data from 4 hospitals to compare the predictive performance between the original and the extended scores, using area under the receiver operating characteristic curve (AUC) and net reclassification improvement (NRI).

**Results:**

In a total of 548 patients, the rates of SICH were 7.3% per the National Institute of Neurological Diseases and Stroke (NINDS) definition, 5.3% per the European-Australasian Cooperative Acute Stroke Study (ECASS) II, and 3.5% per the SITS-Monitoring Study (SITS-MOST). Both scores effectively predicted SICH across all three definitions. The extended score had a higher AUC for SICH per NINDS (0.704 versus 0.624, P = 0.015) and per ECASS II (0.703 versus 0.612, P = 0.016) compared with the SITS SICH risk score. NRI for the extended risk score was 22.3% (P = 0.011) for SICH per NINDS, 21.2% (P = 0.018) per ECASS II, and 24.5% (P = 0.024) per SITS-MOST.

**Conclusions:**

Incorporation of the OCSP classification into the SITS SICH risk score improves risk prediction for post-thrombolysis SICH.

## Background

Thrombolytic therapy with intravenous tissue plasminogen activator (tPA) for acute ischemic stroke increases the risk of symptomatic intracerebral hemorrhage (SICH) [1]. Factors associated with SICH include older age, higher baseline National Institutes of Health Stroke Scale (NIHSS) score, elevated blood glucose, prior antiplatelet use, presence of atrial fibrillation, congestive heart failure, renal impairment, and early ischemic changes on pretreatment brain imaging [2]. Several risk score models incorporating these potential predictors have been constructed to determine the risk of tPA-associated SICH [3-9]. Although the discriminatory abilities of such models appear good, there may be room for further improvement.

Stroke territory may help estimate the risk of post-thrombolysis hemorrhagic transformation because posterior circulation stroke might be associated with a low risk of SICH [10,11]. The concept was first implied in the Hemorrhage After Thrombolysis (HAT) score [4], in which the presence of hypodensity in middle cerebral artery territory on computed tomography (CT) denotes an anterior circulation stroke. No risk prediction models have yet explicitly incorporated stroke territory. However, using neuroimaging study to determine stroke territory may not be applicable or timely in emergency settings. Even magnetic resonance imaging (MRI) detected acute lesions in only 46% of patients with acute ischemic stroke examined within 3 hours after symptom onset [12].

The Oxfordshire Community Stroke Project (OCSP) classification, based on clinical syndromes alone, can predict the site and size of infarct on CT in patients with established or hyperacute ischemic stroke [13,14]. In a previous study [15], we demonstrated that the OCSP classification could help evaluate the risk of post-thrombolysis SICH. We therefore examined whether and how the addition of the OCSP classification could improve current SICH risk scores.

## Methods

### Patients

We analyzed data pooled from 4 hospital-based stroke registries in Taiwan (National Cheng Kung University Hospital, Chi-Mei Medical Center, Chia-Yi Christian Hospital, and Landseed Hospital) [16]. Consecutive stroke patients treated with intravenous tPA within 3 hours of symptom onset between January 2007 and June 2012 were identified. Eligibility for tPA treatment was determined following the guidelines of the American Heart Association [17], and included off-label use (age >80 or NIHSS score >25). Variable dosing in the range of 0.6 to 0.9 mg/kg is recommended according to the current Taiwan guidelines [18]. Different treatment regimens were used in the study sites. At the Chi-Mei Medical Center and the Landseed Hospital, the dose was fixed at 0.9 mg/kg. At the Chia-Yi Christian Hospital, the dose was initially set at 0.9 mg/kg and was changed to 0.8 mg/kg after August 2010. At the National Cheng Kung University Hospital, the dose was initially set at 0.7 mg/kg and was altered to 0.9 mg/kg after July 2009. Stroke severity was assessed by the NIHSS at baseline and at discharge. All patients underwent CT or MRI between 24 and 36 hours after thrombolysis and additional scans in case of clinical deterioration. The official neuroradiology reports were used to determine the presence and size of visible hypodensity on initial head CT scan and the presence or absence of intracerebral hemorrhage (ICH) on follow-up scans. The study protocol was approved by the Landseed Institutional Review Board (IRB), National Cheng Kung University Hospital IRB, Chi-Mei Medical Center IRB, and Chia-Yi Christian Hospital IRB, respectively.

Research coordinators in each site extracted the parts of medical charts recording the neurological symptoms and signs before the administration of intravenous tPA. Two board-certified neurologists (CSW and LCH), who were blinded to neuroimaging findings and risk scores of each patient, independently examined the abstracted records and performed the clinical classification [19]. Patients were classified as having total anterior circulation infarcts (TACI), partial anterior circulation infarcts (PACI), posterior circulation infarcts (POCI), or lacunar infarcts (LACI) based on their maximal neurological deficits before the initiation of thrombolytic therapy. Undetermined stroke syndrome was classified as “uncertain”. Discrepancies were resolved by including a third board-certified neurologist (YCH), or classified as uncertain when all three disagreed with each other.

### Outcome measures

Primary outcome was the occurrence of SICH following intravenous tPA treatment. Because SICH rates are subject to the definition of SICH, we applied three definitions. SICH per the National Institute of Neurological Diseases and Stroke (NINDS) is defined as any neurological worsening (NIHSS ≥1) within 36 hours of tPA administration that is attributed to ICH verified by CT or MRI [1]. SICH per the European-Australasian Cooperative Acute Stroke Study (ECASS) II is defined as any type of ICH on any posttreatment imaging after the start of thrombolysis and a neurological deterioration of ≥4 points on the NIHSS from baseline, or from the lowest value within 7 days, or leading to death [20]. SICH per the Safe Implementation of Thrombolysis in Stroke - Monitoring Study (SITS-MOST) is defined as a local or remote type 2 parenchymal hemorrhage on the 22- to 36-hour post-treatment CT scan associated with a neurological deterioration of ≥4 points on the NIHSS or leading to death [21].

### Calculation of the risk score

We calculated the Safe Implementation of Thrombolysis in Stroke (SITS) SICH risk score (Table 1) for each patient [7]. The decision to apply this particular risk score was based on the facts that it was derived from the largest cohort of patients, applicable in all three definitions of SICH of interest, and has been validated in our study population [16]. The SITS SICH risk score includes 9 variables with different weights (age, baseline NIHSS score, glucose, systolic blood pressure, body weight, time from stroke onset to thrombolysis, history of hypertension, aspirin monotherapy, and combined use of aspirin and clopidogrel) and ranges from 0 to 12 points [7].

**Table 1:** Risk scoring systems of the SITS SICH risk score and the extended risk score

	**No. of points**	
**Risk factor**	**SITS SICH risk score**	**Extended risk score**
Aspirin + clopidogrel	3	3
Aspirin monotherapy	2	2
NIHSS ≥13	2	2
NIHSS 7-12	1	1
Blood glucose ≥180 mg/dL	2	2
Age ≥72 y	1	1
Systolic blood pressure ≥146 mm Hg	1	1
Weight ≥95 kg	1	1
Onset-to-treatment time ≥180 min	1	1
History of hypertension	1	1
Total anterior circulation infarct	N/A	3
Maximal total points	12	15*

N/A indicates not applicable; SICH: symptomatic intracerebral hemorrhage; SITS: the Safe Implementation of Thrombolysis in Stroke.

*Patients with posterior circulation infarcts were assigned an extended risk score of zero, irrespective of their original SITS SICH risk scores.

We constructed an extended SITS SICH risk score (Table 1) by incorporating an OCSP classification component. In light of a finding that TACI increased sixfold the risk of SICH per ECASS II (after adjustment for age, glucose, and antiplatelet use before admission) [15], initially we assigned 3 points to TACI and 0 point to PACI or LACI in the OCSP classification component. Furthermore, considering the low risk of SICH in posterior circulation stroke [10,11], patients with POCI were assigned an extended risk score of zero, irrespective of their original SITS SICH risk scores. After examining the discrimination ability of different weighting of TACI using the area under the receiver operating characteristic curve (AUC) (Additional file 1: Table S1), we eventually constructed the 15-point extended risk score as described above.

### Statistical analysis

Continuous variables were summarized as mean ± SD or median (interquartile range), and categorical variables as counts and percentages. We performed separate univariate logistic regressions for the original and the extended risk score to determine the odds ratios per point increase of the score. The model fit was judged by the Hosmer–Lemeshow goodness-of-fit statistic. The discriminatory ability of the extended score was evaluated by the AUC, and compared using the DeLong method [22]. Bootstrap resampling (1000 replicates) was used to estimate confidence intervals for the AUC.

We examined risk reclassification from strata of the SITS SICH risk score to those of the extended risk score by cross tabulation. For practical purposes, we stratified the SITS SICH risk score into 3 categories: low (0 to 2 points), average (3 to 5 points), and elevated risk (≥6 points). The same cutoffs were used in the SITS SICH risk score study [7], but we collapsed strata of 6 to 8 points and ≥9 points into one group. To align the thresholds for defining risk categories, the extended risk score was categorized into low (0 to 3 points), average (4 to 7 points), and elevated risk (≥8 points). We used the net reclassification improvement (NRI), calculated as [proportion of all SICH events reclassified at higher risk – proportion reclassified at lower risk] – [proportion of all nonevents reclassified at higher risk – proportion reclassified at lower risk] [23], to assess the incremental effect of adding the OCSP classification component. Two tailed P values <0.05 were considered statistically significant. Statistical analyses were performed using MedCalc 12.5.0 (MedCalc Software bvba, Ostend, Belgium) or Stata 11 (StataCorp, College Station, Texas).

## Results

A total of 548 thrombolyzed patients were included in the study. Table 2 shows the characteristics. The rates of SICH were 7.3% per the NINDS definition, 5.3% per ECASS II, and 3.5% per SITS-MOST. The agreement of the OCSP classification was moderate (κ = 0.583) between the two initial assessors (Additional file 1: Table S2).

**Table 2:** Characteristics of the study patients

	**n = 548**
Demographics	
Age, mean (SD)	67 (12)
Male, n (%)	345 (63.0)
Medical history, n (%)	
Hypertension	406 (74.1)
Diabetes mellitus	177 (32.3)
Hyperlipidaemia	303 (55.3)
Atrial fibrillation	169 (30.8)
Congestive heart failure	37 (6.8)
Prior stroke/TIA	116 (21.2)
Current smoking	183 (33.4)
Antiplatelets	129 (23.5)
Warfarin	9 (1.6)
Clinical data	
Baseline NIHSS score, median (IQR)	13 (8–20)
Body weight, mean (SD), kg	65 (13)
Systolic blood pressure, mean (SD), mm Hg	161 (30)
Glucose, mean (SD), mmol/L	8.49 (3.72)
Platelet count, mean (SD), ×10^9^/L	215 (70)
Actual tPA dose, median (IQR), mg/kg	0.86 (0.75–0.91)
OTT, median (IQR), min	125 (100–155)
OCSP classification, n (%)	
TACI	207 (37.8)
PACI	162 (29.6)
POCI	48 (8.8)
LACI	111 (20.3)
Uncertain	20 (3.6)

IQR indicates interquartile range; LACI: lacunar infarct; NIHSS: National Institutes of Health Stroke Scale; OCSP: Oxfordshire Community Stroke Project; OTT: onset-to-treatment time; PACI: partial anterior circulation infarcts; POCI: posterior circulation infarcts; SD: standard deviation; TACI: total anterior circulation infarcts; TIA: transient ischemic attack; tPA: tissue-type plasminogen activator.

Both the SITS SICH risk score and the extended risk score reasonably predicted the occurrence of SICH and were well calibrated (Table 3). The AUCs were higher for the extended risk score than for the SITS SICH risk score across all definitions of SICH, with significant differences per NINDS (P = 0.015) and per ECASS II (P = 0.016). Reclassification of patients improved with an NRI of 22.3% (P = 0.011) for SICH per NINDS, 21.2% (P = 0.018) per ECASS II, and 24.5% (P = 0.024) per SITS-MOST (Additional file 1: Table S3 to S5). Given the designated risk strata, the SITS SICH risk score categorized 19% of patients at low risk, 66% at average risk, and 15% at elevated risk, whereas the corresponding values by the extended risk score were 37%, 44%, and 19%.

**Table 3:** Comparison of prediction performance between the SITS SICH risk score and the extended risk score

	**Odds ratio (95% CI) per point**	**Hosmer-Lemeshow statistic**	**P**	**AUC (95% CI)**	**Difference between areas**	**P**	**Net reclassification improvement**	**P**
SICH per NINDS								
SITS SICH risk score	1.35 (1.11-1.64)	1.72	0.887	0.624 (0.533-0.714)	-	-	-	-
Extended risk score	1.30 (1.15-1.46)	4.30	0.745	0.704 (0.618-0.791)	0.081	0.015	22.3%	0.011
SICH per ECASS II								
SITS SICH risk score	1.34 (1.08-1.68)	3.25	0.661	0.612 (0.503-0.721)	-	-	-	-
Extended risk score	1.30 (1.13-1.49)	4.23	0.752	0.703 (0.611-0.796)	0.091	0.016	21.2%	0.018
SICH per SITS-MOST								
SITS SICH risk score	1.49 (1.14-1.94)	2.10	0.835	0.678 (0.563-0.793)	-	-	-	-
Extended risk score	1.33 (1.12-1.58)	6.45	0.488	0.723 (0.622-0.824)	0.044	0.293	24.5%	0.024

AUC indicates area under the receiver operating characteristic curve; CI: confidence intervals; ECASS: The European-Australasian Cooperative Acute Stroke Study; NINDS: National Institute of Neurological Disorders and Stroke; SICH: symptomatic intracerebral hemorrhage; SITS-MOST: the Safe Implementation of Thrombolysis in Stroke - Monitoring Study.

## Discussion

We demonstrated that incorporation of the OCSP classification into the SITS SICH risk score significantly improved the performance in predicting SICH in our study population. The extended risk score reasonably predicted SICH across the three definitions with good calibration. A substantial proportion of patients without SICH were reclassified into lower risk category (NRI for these patients was about 14% across the three definitions of SICH). Overall, the extended risk score moved an additional 18% (37% minus 19%) of patients to the low risk category, and 46% (168 of 362) patients with average risk (3 to 5 points according to the SITS SICH risk score) were reclassified (Additional file 1: Table S3 to S5).

The improved discriminatory ability of the extended risk score is clinically useful and might facilitate thrombolytic treatment in acute ischemic stroke. Notably, many emergency physicians were unwilling to provide thrombolysis for acute ischemic stroke in fear of SICH [24]. In Taiwan, perceived risk of SICH among emergency physicians, neurologists, and patients was a major barrier to implement thrombolytic treatment [25]. Risk prediction models that could correctly identify patients at low SICH risk after thrombolysis are of great benefit to stroke patients because they help remove the psychological barriers to administering tPA.

Studies show that patients with posterior circulation stroke are unlikely to develop SICH or hemorrhagic transformations [10,11]. Therefore in our study the patients with POCI were assigned an extended risk score of zero. A potential mechanism underlying the low incidence of SICH in posterior circulation stroke is the infrequent permeability derangements detected on pretreatment MRI [26]. Permeability derangements, which indicate blood–brain barrier disruption in ischemic fields, increased the propensity for hemorrhagic transformation in stroke patients treated with either recanalization therapy or conservative care [27]. Another possible mechanism is better collaterals in the territory of the posterior cerebral artery than that of the middle cerebral artery [28]. Patients with good collateral circulation might be less vulnerable to SICH after recanalization therapy [29,30]. Additionally, the size of ischemic brain tissue was associated with SICH after intravenous thrombolysis [31,32]. The small lesion volume in infratentorial strokes as compared to supratentorial strokes might partly explain the low incidence of SICH in patients with POCI.

Because the volume of the affected brain tissue is generally correlated with clinical stroke severity, models for predicting post-thrombolysis SICH usually included the NIHSS or the less commonly used Canadian Neurological Scale as a predictor [3-9]. However, patients with right hemisphere strokes may have a low NIHSS score despite a substantial lesion volume [33]. Consequently, patients with right hemisphere nonlacunar strokes would have a higher risk of post-thrombolysis hemorrhage than those with left hemisphere strokes and similar NIHSS scores [34]. In contrast, the OCSP classification in the early hours of ischemic stroke correlated well with infarct size [14]. Without the assessment bias between hemispheres, the OCSP classification complements the NIHSS score in predicting SICH risk.

Although the OCSP classification generally corresponded well to the radiological findings, the sensitivity and specificity were different among the four OCSP categories. Using lesion topography on diffusion-weighted MRI as the diagnostic standard, the likelihood of correct classification was low for PACI and LACI, whereas high for TACI and POCI [35]. Therefore, we did not differentiate patients with PACI from those with LACI in designing the extended risk score. Theoretically, a prediction model combining imaging findings and clinical factors could improve the prediction of SICH. A recent study indicated that the blood Sugar, Early infarct signs, hyperDense cerebral artery sign, Age, and NIHSS (SEDAN) score [6] had the highest predictive power among the existing risk prediction scores [36]. Our previous study also found that the HAT score performed well [16]. However, the interpretation of early infarct signs or hyperdense cerebral artery sign requires considerable radiological expertise, which may not be feasible in settings where neuroradiologists are not available on a 24/7 basis [37]. In addition to indicating an anterior circulation infarct, the appearance of early infarct signs on CT also hints at longer elapsed time since stroke onset [38]. The inclusion of total anterior circulation infarct and the onset-to-treatment time, which is a component of the SITS SICH risk score, in our revised risk score offers a clinical counterpart to the radiological findings of early infarct signs. Although the SITS SICH risk score in conjunction with the OCSP classification seems more complex, it provides an alternative when the local radiological expertise is limited. In particular, the OCSP classification could be easily and reliably determined using a standard symptom list [39].

Methods of model assessment should depend on the intended use of the model. Intravenous thrombolysis should not be withheld for an otherwise eligible patient simply because of high anticipated risk of SICH. The demonstrated improvement in classification performance in the extended risk score should be used only to better comprehend the risk associated with thrombolytic therapy. In particular, reclassification performance is sensitive to the number of clinically relevant risk categories, and the OCSP classification renders applying the risk scores largely unnecessary in patients with POCI.

Awareness of the applications and potential limitations of the risk scores will aid the clinicians in daily clinical decision making. Patients and their family could be better informed of the risk of SICH before making a shared treatment decision with their physicians. Those at high risk of SICH may benefit from more intensive monitoring, such as blood pressure and blood glucose. Another potential use of the risk scores is for case-mix adjustment in light of the fact that post-thrombolysis SICH might be used to measure performance of acute stroke care [40].

Our study has limitations. First, the OCSP syndromes were assessed based on medical records, rather than personal examination. Although the interrater agreement in the clinical classification of syndromes was moderate, the accuracy of classification might be compromised. Further studies with prospective OCSP classification are needed to confirm the clinical implications of our findings. Second, the number of SICH events was small, which precluded multivariable analyses, such as reweighting the predictors of the SITS SICH risk score. The limited number of events might also explain the failure to show superior discrimination (higher AUC) of the extended risk score in predicting SICH per the SITS-MOST definition. Third, a proportion of our patients were treated with a lower dose of intravenous tPA. Whether the prediction performance of risk models is subject to dosage remains to be explored. Finally, our study should be viewed as hypothesis generating. Potential patient selection bias could impact the model performance. Further validation is needed to strengthen generalizability of our findings.

## Conclusions

Incorporating the OCSP classification of stroke syndromes to the SITS SICH risk score could improve the risk prediction for post-thrombolysis SICH. Further studies to evaluate the performance of the extended risk score on large cohorts are warranted.

## Competing interests

The authors declare that they have no competing interests.

## Authors’ contributions

SFS, conception and design, analysis and interpretation of data, drafting the article; SCCC, analysis and interpretation of data, revising the manuscript critically for important intellectual content; HJL, conception and design, analysis and interpretation of data, revising the manuscript critically for important intellectual content; CHC, conception and design, analysis and interpretation of data, revising the manuscript critically for important intellectual content; MCT, analysis and interpretation of data, revising the manuscript critically for important intellectual content; CSW, analysis and interpretation of data, revising the manuscript critically for important intellectual content; YCH, analysis and interpretation of data, revising the manuscript critically for important intellectual content; LCH, analysis and interpretation of data, revising the manuscript critically for important intellectual content. YWC, conception and design, analysis and interpretation of data, revising it critically for important intellectual content, final approval of the version to be published. All authors read and approved the final manuscript.

## Pre-publication history

The pre-publication history for this paper can be accessed here:

http://www.biomedcentral.com/1471-2377/14/39/prepub

## Supplementary Material

Additional file 1: Table S1 AUCs for prediction of SICH calculated for the extended SITS SICH risk score with various weighting on TACI. **Table S2.** Interrater agreement of the Oxfordshire Community Stroke Project classification of stroke syndromes. **Table S3.** Reclassification table comparing risk strata of SICH per NINDS. **Table S4.** Reclassification table comparing risk strata of SICH per ECASS II. **Table S5.** Reclassification table comparing risk strata of SICH per SITS-MOST. Click here for file
