# Supplementary material for: Oxfordshire community stroke project classification improves prediction of post-thrombolysis symptomatic intracerebral hemorrhage
Source: BMC Neurol. 2014 Mar 1;14:39. doi: 10.1186/1471-2377-14-39 (PMC3941257; doi:10.1186/1471-2377-14-39)
Supplement: Additional file 1: Table S1 — AUCs for prediction of SICH calculated for the extended SITS SICH risk score with various weighting on TACI. Table S2. Interrater agreement of the Oxfordshire Community Stroke Project classification of stroke syndromes. Table S3. Reclassification table comparing risk strata of SICH per NINDS. Table S4. Reclassification table comparing risk strata of SICH per ECASS II. Table S5. Reclassification table comparing risk strata of SICH per SITS-MOST. [file 1471-2377-14-39-S1.pdf]

ADDITIONAL FILE 1

Table 1 AUCs for prediction of SICH calculated for the extended SITS SICH risk score with various weighting on TACI.

	AUC			
Points allocated to TACI	1	2	3	4
SICH per NINDS	0.674	0.695	0.704	0.706
SICH per ECASS II	0.672	0.692	0.703	0.706
SICH per SITS-MOST	0.715	0.720	0.723	0.721

AUC indicates area under the receiver operating characteristic curve; ECASS: The European-Australasian Cooperative Acute Stroke Study; NINDS: National Institute of Neurological Disorders and Stroke; SICH: symptomatic intracerebral hemorrhage; SITS-MOST: the Safe Implementation of Thrombolysis in Stroke - Monitoring Study; TACI: total anterior circulation infarcts.

Table 2 Interrater agreement of the Oxfordshire Community Stroke Project classification of stroke syndromes.

Neurologist 2	Neurologist 1					
	TACI	PACI	POCI	LACI	Uncertain	Total
TACI	167	13	9	0	0	189 (34.5%)
PACI	29	119	14	1	0	163 (29.7%)
POCI	14	3	27	0	0	44 (8.0%)
LACI	4	65	11	69	0	149 (27.2%)
Uncertain	0	0	0	0	3	3 (0.5%)
Total	214 (39.1%)	200 (36.5%)	61 (11.1%)	70 (12.8%)	3 (0.5%)	548

LACI indicates lacunar infarcts; PACI: partial anterior circulation infarcts; POCI: posterior circulation infarcts; TACI: total anterior circulation infarcts.

Table 3 Reclassification table comparing risk strata of SICH per NINDS.

SITS SICH risk score	Extended risk score: 0% to <4% (0-3 points)	4% to 11% (4-7 points)	>11% (≥8 points)	Total	Risk reclassification: Lower	Higher	Total
0% to <4% (0-2 points)							
At risk	85	18	0	103	-	18	18
SICH	2	1	0	3	-	1	1
No SICH	83	17	0	100	-	17	17
Observed risk	2.4%	5.6%	-	2.9%	-	-	-
4% to 11% (3-5 points)							
At risk	113	194	55	362	113	55	168
SICH	3	16	6	25	3	6	9
No SICH	110	178	49	337	110	49	159
Observed risk	2.7%	8.2%	10.9%	6.9%	-	-	-
>11% (≥6 points)							
At risk	4	28	51	83	32	-	32
SICH	0	1	11	12	1	-	1
No SICH	4	27	40	71	31	-	31
Observed risk	0.0%	3.6%	21.6%	14.5%	-	-	-
Total							
At risk	202	240	106	548	145	73	218
SICH	5	18	17	40	4	7	11
No SICH	197	222	89	508	141	66	207
Observed risk	2.5%	7.5%	16.0%	7.3%	-	-	-

NINDS indicates National Institute of Neurological Disorders and Stroke; SICH: symptomatic intracerebral hemorrhage; SITS: the Safe Implementation of Thrombolysis in Stroke.

Red background refers to an increase in risk stratum; blue background to a decrease in risk stratum.

Table 4 Reclassification table comparing risk strata of SICH per ECASS II.

SITS SICH risk score	Extended risk score: 0% to <3% (0-3 points)	Extended risk score: 3% to 8% (4-7 points)	Extended risk score: >8% (≥8 points)	Total	Risk reclassification: Lower	Risk reclassification: Higher	Risk reclassification: Total
**0% to <3% (0-2 points)**							
At risk	85	18	0	103	-	18	18
SICH	1	1	0	2	-	1	1
No SICH	84	17	0	101	-	17	17
Observed risk	1.2%	5.6%	-	1.9%	-	-	-
**3% to 8% (3-5 points)**							
At risk	113	194	55	362	113	55	168
SICH	2	13	3	18	2	3	5
No SICH	111	181	52	344	111	52	163
Observed risk	1.8%	6.7%	5.5%	5.0%	-	-	-
**>8% (≥6 points)**							
At risk	4	28	51	83	32	-	32
SICH	0	0	9	9	0	-	0
No SICH	4	28	42	74	32	-	32
Observed risk	0.0%	0.0%	17.6%	10.8%	-	-	-
**Total**							
At risk	202	240	106	548	145	73	218
SICH	3	14	12	29	2	4	6
No SICH	199	226	94	519	143	69	212
Observed risk	1.5%	5.8%	11.3%	5.3%	-	-	-

ECASS indicates The European-Australasian Cooperative Acute Stroke Study; SICH: symptomatic intracerebral hemorrhage; SITS: the Safe Implementation of Thrombolysis in Stroke.

Red background refers to an increase in risk stratum; blue background to a decrease in risk stratum.

Table 5 Reclassification table comparing risk strata of SICH per SITS-MOST.

	Extended risk Score				Risk reclassification		
SITS SICH risk score	0% to <2% (0-3 points)	2% to 5% (4-7 points)	>5% (≥8 points)	Total	Lower	Higher	Total
0% to <2% (0-2 points)							
At risk	85	18	0	103	-	18	18
SICH	0	1	0	1	-	1	1
No SICH	85	17	0	102	-	17	17
Observed risk	0.0%	5.6%	-	1.0%	-	-	-
2% to 5% (3-5 points)							
At risk	113	194	55	362	113	55	168
SICH	1	8	2	11	1	2	3
No SICH	112	186	53	351	112	53	165
Observed risk	0.9%	4.1%	3.6%	3.0%	-	-	-
>5% (≥6 points)							
At risk	4	28	51	83	32	-	32
SICH	0	0	7	7	0	-	0
No SICH	4	28	44	76	32	-	32
Observed risk	0.0%	0.0%	13.7%	8.4%	-	-	-
Total							
At risk	202	240	106	548	145	73	218
SICH	1	9	9	19	1	3	4
No SICH	201	231	97	529	144	70	214
Observed risk	0.5%	3.8%	8.5%	3.5%	-	-	-

SICH indicates symptomatic intracerebral hemorrhage; SITS-MOST: the Safe Implementation of Thrombolysis in Stroke - Monitoring Study.

Red background refers to an increase in risk stratum; blue background to a decrease in risk stratum.
